# Patient genetics is linked to chronic wound microbiome composition and healing

**DOI:** 10.1371/journal.ppat.1008511

**Published:** 2020-06-18

**Authors:** Craig D. Tipton, Randall D. Wolcott, Nicholas E. Sanford, Clint Miller, Gita Pathak, Talisa K. Silzer, Jie Sun, Derek Fleming, Kendra P. Rumbaugh, Todd D. Little, Nicole Phillips, Caleb D. Phillips

**Affiliations:** 1 Department of Biological Sciences, Texas Tech University, Lubbock, Texas, United States of America; 2 RTL Genomics, Lubbock, Texas, United States of America; 3 Southwest Regional Wound Care Center, Lubbock, Texas, United States of America; 4 Microbiology, Immunology & Genetics, University of North Texas Health Science Center, Fort Worth, Texas, United States of America; 5 Department of Surgery, Texas Tech University Health Sciences Center, Lubbock, Texas, United States of America; 6 Burn Center of Excellence, Texas Tech University Health Sciences Center, Lubbock, Texas, United States of America; 7 Department of Educational Psychology, Texas Tech University, Lubbock, Texas, United States of America; 8 Optentia Research Focus Area, North West University, Vanderbijlpark, South Africa; 9 Natural Science Research Laboratory, Texas Tech University, Lubbock, Texas, United States of America; University of North Carolina at Chapel Hil, UNITED STATES

## Abstract

The clinical importance of microbiomes to the chronicity of wounds is widely appreciated, yet little is understood about patient-specific processes shaping wound microbiome composition. Here, a two-cohort microbiome-genome wide association study is presented through which patient genomic loci associated with chronic wound microbiome diversity were identified. Further investigation revealed that alternative *TLN2* and *ZNF521* genotypes explained significant inter-patient variation in relative abundance of two key pathogens, *Pseudomonas aeruginosa* and *Staphylococcus epidermidis*. Wound diversity was lowest in *Pseudomonas aeruginosa* infected wounds, and decreasing wound diversity had a significant negative linear relationship with healing rate. In addition to microbiome characteristics, age, diabetic status, and genetic ancestry all significantly influenced healing. Using structural equation modeling to identify common variance among SNPs, six loci were sufficient to explain 53% of variation in wound microbiome diversity, which was a 10% increase over traditional multiple regression. Focusing on *TLN2*, genotype at *rs8031916* explained expression differences of alternative transcripts that differ in inclusion of important focal adhesion binding domains. Such differences are hypothesized to relate to wound microbiomes and healing through effects on bacterial exploitation of focal adhesions and/or cellular migration. Related, other associated loci were functionally enriched, often with roles in cytoskeletal dynamics. This study, being the first to identify patient genetic determinants for wound microbiomes and healing, implicates genetic variation determining cellular adhesion phenotypes as important drivers of infection type. The identification of predictive biomarkers for chronic wound microbiomes may serve as risk factors and guide treatment by informing patient-specific tendencies of infection.

## Author summary

Chronic, or non-healing, wounds represent a costly burden to patients, and bacterial infection of wounds is an important driver of chronicity. A variety of bacterial species often occur in chronic wounds, but it is unknown why certain species are observed in some wound infections and not others. In this study, genetic variation of wound clinic patients was compared to the bacteria observed in their infected wounds. Through these comparisons, genetic variation in the *TLN2* and *ZNF521* genes was found to be associated with both the number of bacteria observed in wounds and the abundance of common pathogens (primarily *Pseudomonas aeruginosa* and *Staphylococcus epidermidis*). Moreover, *Pseudomonas* infected wounds were found to have fewer species present and wounds with fewer species were slower to heal. Furthermore, patient genes associated with microbiomes commonly encode proteins known to be important for cellular structures important to healing and to which bacteria directly interact. Experimental investigation of one such gene, *TLN2*, identified genotype-dependent differences in the expression of functionally different versions of *TLN2* that is hypothesized to shape differences in cellular adhesion structures. Finally, a new statistical approach is presented in which patient biomarkers are used to predict the number of species observed during infection. Overall, our results describe how patient genetic variation influence the types of bacteria likely to infect an individual as well as influence healing.

## Introduction

Chronic wounds, defined as wounds failing to show signs of healing within three weeks, are a significant and increasing burden on the health care system, resulting in several billion dollars in annual health care costs in the United States alone [1]. Elderly individuals and diabetics have a significantly increased likelihood of developing chronic wounds [2]. Although the initial emergence of wounds has multiple etiologies, the development of chronicity is heavily influenced by the colonization of bacteria [3, 4], and also fungi [5], which are thought to interact through community ecological processes to shape individual wound microbiomes and stall healing [4].

Multiple exploratory studies have helped to define characteristics of wound microbiomes and highlighted relevance to healing outcomes. Wound microbiomes are compositionally distinct from contra-lateral healthy skin microbiomes and species that are observed in both exhibit altered relative abundances, likely reflecting a switch from commensalism to opportunistic pathogenicity [6]. While typically not as diverse as healthy skin microbiomes [6, 7], wound microbiomes are normally polymicrobial; a study based on approximately 3,000 wound microbiomes reported a median of 6 bacterial species per wound occurring at greater than 1% relative abundance. Wound microbiome composition is thought to be influenced by ecological processes and the resulting polymicrobial infections can exhibit synergistic effects such as enhanced tolerance to antimicrobials [8–10]. Furthermore, chronic wound microbiomes can be dynamic through time [11] with bacterial diversity increasing at resolution of infection [12], and stable microbial communities being correlated with delayed healing [4, 12]. Despite these findings, the clinical importance of many taxa and the diversity of community compositions observed among wound infections remains unclear.

A developing body of literature points to host genetics as an important determinant of composition of host-associated microbiomes. At a broad level the observation that the unique physiologies of different body sites, which are genetically determined, support distinct microbiome communities specifies a degree of host genetic determination [7]. However, the amount of genetic variation within populations that influence inter-individual differences in microbiomes at body sites is not known [13]. Independent twin studies based on sampling from different countries have reported a consensus view that gut microbiome composition has a heritable component [14, 15]. A few recent microbiome genome-wide association studies (mbGWAS) have identified specific loci that significantly associate with bacterial species (see reviews by Awany *et al*. [16] and Goodrich *et al*. [13]). The currently limited understanding about how host genetics influence microbiomes comes primarily from studies of healthy participants, and even less is known about how host genetics influence microbiomes in disease states.

In the context of infection, candidate gene approaches have identified differences at innate immunity-associated loci between subjects that develop skin infections and healthy controls [17], yet how host genetics influences types of infection is unknown. A recent mbGWAS identified loci associated with the occurrence of *Staphylococcus aureus* in healthy sinuses [18], loci which may be relevant to infection given the opportunistic pathogenicity of *S*. *aureus*. Other work reported a locus segregating among diabetic subjects who either did, or did not, develop a foot ulcer over the course of study [19]. The rationale for the current study was that given the previously reported diversity and variability in wound microbiomes, paired with the emerging view of how host genetic factors shape microbiomes, inter-patient genetic variability may be important in shaping wound microbiome composition. The identification of loci shaping the microbiome of chronic wounds is expected to inform not only mechanistic details of host-microbiota interactions but will guide the identification of predictive biomarkers and potential therapeutic targets. A set of related predictions were assessed in the current study. Specifically, that chronic wound microbiome composition significantly associates with patient loci, that identified loci select for specific species which influence microbiome composition, that compositional variance is related to healing variance, and that identified loci can be used to inform a predictive model.

## Results

### Loci with non-random associations to wound microbiome diversity and composition are identified through mbGWAS

During patients’ initial clinical visit for chronic wound care, wound debridement was collected, homogenized, and used to characterize individual wound microbiome composition with 16S rRNA gene amplicon sequencing. Buccal swabs were also collected for each patient and processed for genotyping, initially at 665,608 single nucleotide polymorphisms (SNPs). Following quality control measures [20], the exploratory cohort narrowed to 79 individuals with a total 317,553 SNPs (99.0% call rate). Genotypic associations with wound microbiome alpha diversity (Hill_1_ diversity) were conducted naively to all available SNPs [21], controlling for diabetes, age, sex, and population stratification (S1 Fig, S2 Fig). Eight SNPs were identified to have suggestive associations with wound alpha diversity (p < 1.57 x 10^−5^; S2 Fig, S1 Table) and were subsequently considered as candidate loci.

**Fig 1:**
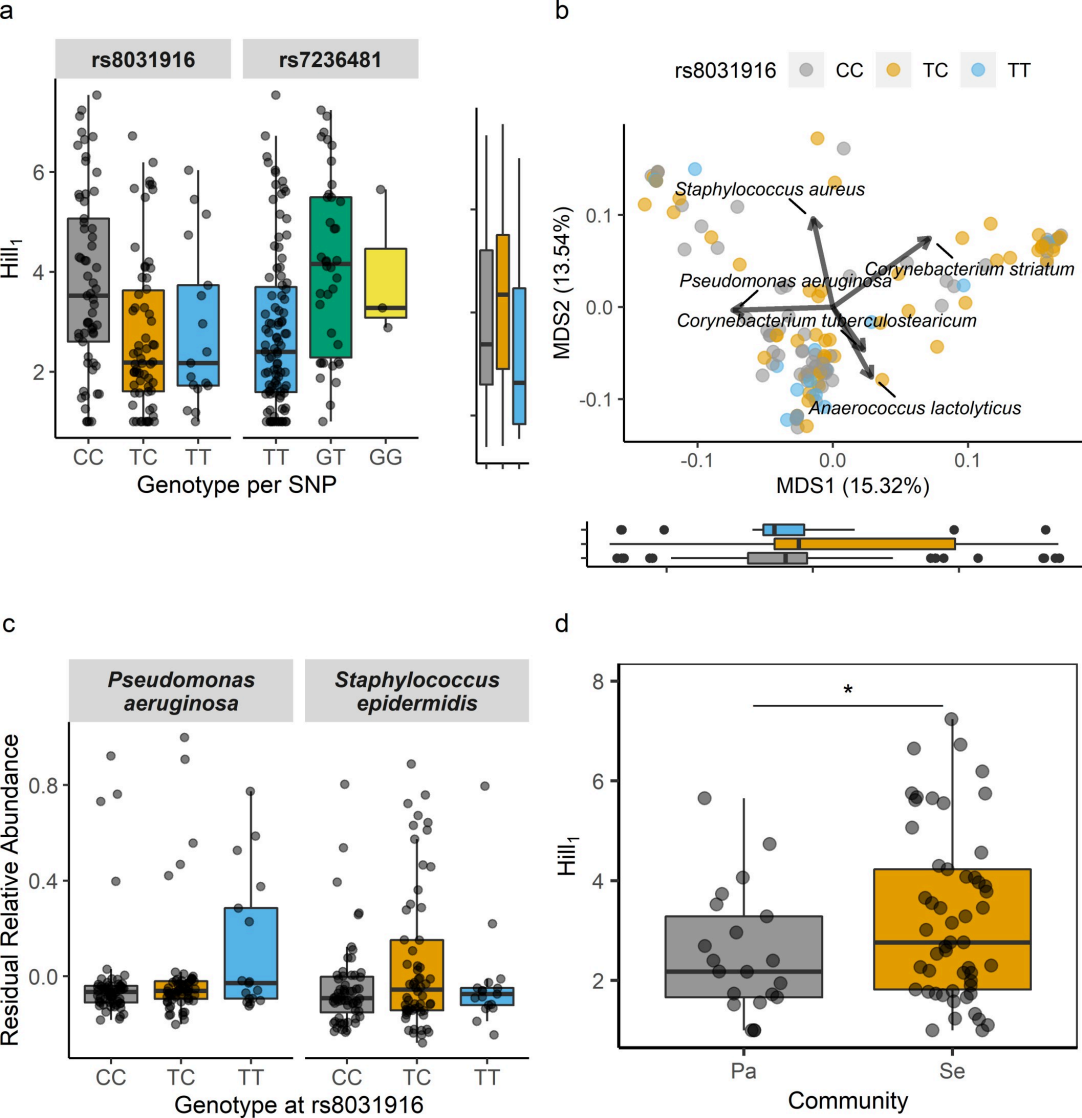
Patient genotype influences on different aspects of the chronic wound microbiome. **a**, Boxplot of Hill_1_ diversity as a function of genotype at *rs8031916* (*TLN2*) and *rs7236481* (*ZNF521*), each explaining significant components of variation in the two-cohort mbGWAS. **b**, Distance-based redundancy analysis based on Bray-Curtis dissimilarities with relative abundance species effects indicated by vectors. *rs8031916* significantly explained beta diversity (p < 0.01). The distribution of genotypes at *rs8031916* across MDS1 and MDS2 are illustrated by boxplots. **c**, Boxplot illustrating how residual relative abundances of *P*. *aeruginosa* and *S*. *epidermidis* were significantly explained by *rs8031916* (p < 0.05 for each). **d**, Communities in which *P*. *aeruginosa* (Pa) was present were significantly less diverse than communities in which *S*. *epidermidis* occurred (p < 0.05).

Following exploratory analysis, an experimental cohort of an additional 85 patients were recruited with 316,671 typed SNPs (99.8% call rate) passing QC. The eight candidate SNPs were tested *a priori* for linear associations with wound alpha diversity. Among these comparisons genotype distributions at *rs8031916* (p = 0 .01) and *rs7236481* (p = 0.01) remained significantly associated with wound alpha diversity (Fig 1A). *rs8031916* is an intronic variant within Talin 2 (*TLN2*, S3 Fig), and *rs7236481* is an intronic variant within Zinc Finger 521 (*ZNF521*; S4 Fig).

Because microbiome alpha diversity and composition are related, how patient genotype at *rs8031916* or *rs7236481* explained variance in patient wound microbiome composition was next quantified. Permutational analysis of variance that incorporated samples from both cohorts, covariates and beta diversity summarized as Bray-Curtis dissimilarities identified a significant but small component of community compositional variation that was explained by *rs8031916* (Fig 1B, F = 2.04, R^2^ = 0.028, p = 0.006) but not *rs7236481* (F = 1.14, R^2^ = 0.016, p = 0.286). Age-related effects on wound microbiome composition were also significant (F = 2.13, R^2^ = 0.015, p = 0.014).

### Relative abundance of bacterial species is explained by patient genotype

The identification of significant associations of *rs8031916* and *rs7236481* with alpha diversity (Fig 1A), and *rs8031916* with beta diversity led to the prediction that patient genotype would explain variance in the relative abundance of certain bacterial lineages. The rationale for this prediction was that alternative patient genotypes may result in phenotypic differences selecting for specific bacterial lineages through host-microbial interactions. Moreover, community interaction among bacterial lineages may influence the diversity and composition of wound microbiomes and would explain the association of patient genotype with diversity. Controlling for covariates and considering bacterial species which were present in at least 10% of wounds resulted in the identification that *Pseudomonas aeruginosa* (p = 0.036) and *Staphylococcus epidermidis* (p = 0.034) relative abundances were significantly explained by *rs8031916* genotype (Fig 1C). In contrast, *rs7236481* genotype marginally explained *S*. *lugdunensis* (p = 0.058) and *Finegoldia magna* (p = 0.068). However, *rs7236481* exhibited a sample size inequality due to a rare genotype (GG, n = 3, i.e. 2% of patients). Repeating the analysis to the exclusion of the rare genotype resulted in additional explanation of *S*. *lugdunensis* relative abundance variance (p = 0.022). Additionally, age was found to be a significant predictor of *S*. *epidermidis* (p = 0.044), *Corynebacterium tuberculostearicum* (p = 0.024), and *Streptococcus agalactiae* (p = 0.024).

**Fig 2:**
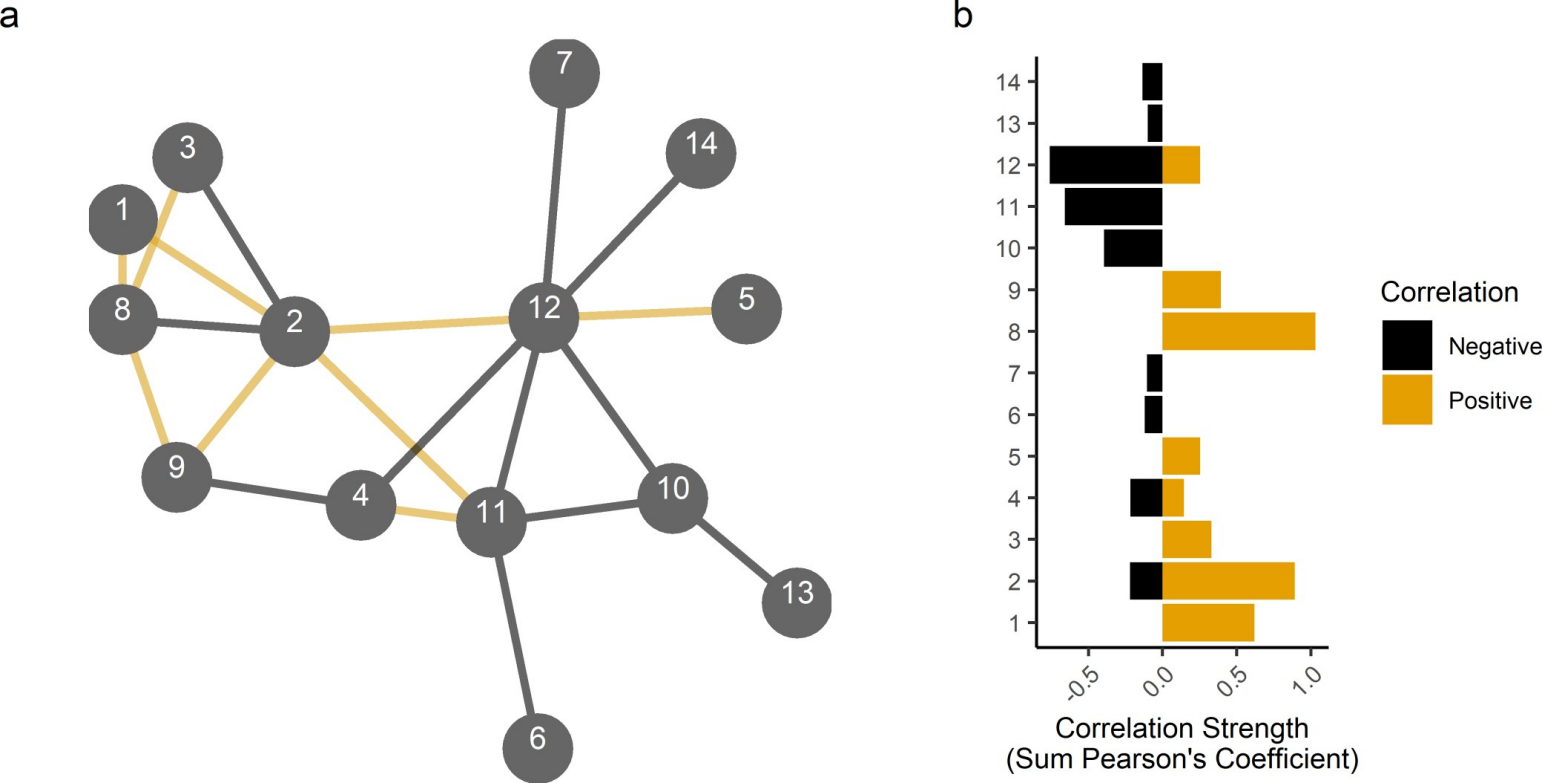
Interspecific interactions inferred from relative abundance correlations. **a,** Interaction network with positive associations denoted by black edges and negative associations denoted by orange edges. Species included had a study-wide average relative abundance greater than 5% and had at least one correlation greater than or equal to r = 0.10. **b,** Barplot of summed positive and negative correlations for each species. Species key b: 1) *Anaerococcus hydrogenalis*, 2) *Anaerococcus lactolyticus*, 3) *Anaerococcus prevotii*, 4) *Anaerococcus vaginalis*, 5) *Corynebacterium tuberculostearicum*, 6) *Finegoldia magna*, 7) *Fusobacterium nucleatum*, 8) *Peptoniphilus harei*, 9) *Porphyromonas levii*, 10) *Pseudomonas aeruginosa*, 11) *Staphylococcus aureus*, 12) *Staphylococcus epidermidis*, 13) *Staphylococcus lugdunensis*, 14) *Streptococcus agalactiae*.

The relationship of patient genotype with wound microbiome diversity, composition and species relative abundances was further investigated by focusing on the *rs8031916* locus and assessing how wound microbiome diversity varied depending on whether wounds were colonized by *P*. *aeruginosa* or *S*. *epidermidis*. Using a Welch’s t-test significantly higher diversity was observed for communities containing *S*. *epidermidis* as compared to those containing *P*. *aeruginosa* (Fig 1D, t = 2.1, df = 49.3, p = 0.02). Furthermore, *P*. *aeruginosa* infected wounds were significantly less diverse as compared to all other wounds (t = 2.3, df = 32.5, p = 0.01). Differences in diversity depending on species’ presence indicated community interaction dynamics may inform the relationship of patient genotype with diversity, composition and species relative abundance. Canonical Pearson correlation and species interaction network construction identified that *P*. *aeruginosa*, *S*. *epidermidis and S*. *aureus* interactions were among the most strongly negative inferred interactions (Fig 2).

### Patient genotype-associated wound microbiome parameters explain healing outcome

**Fig 3:**
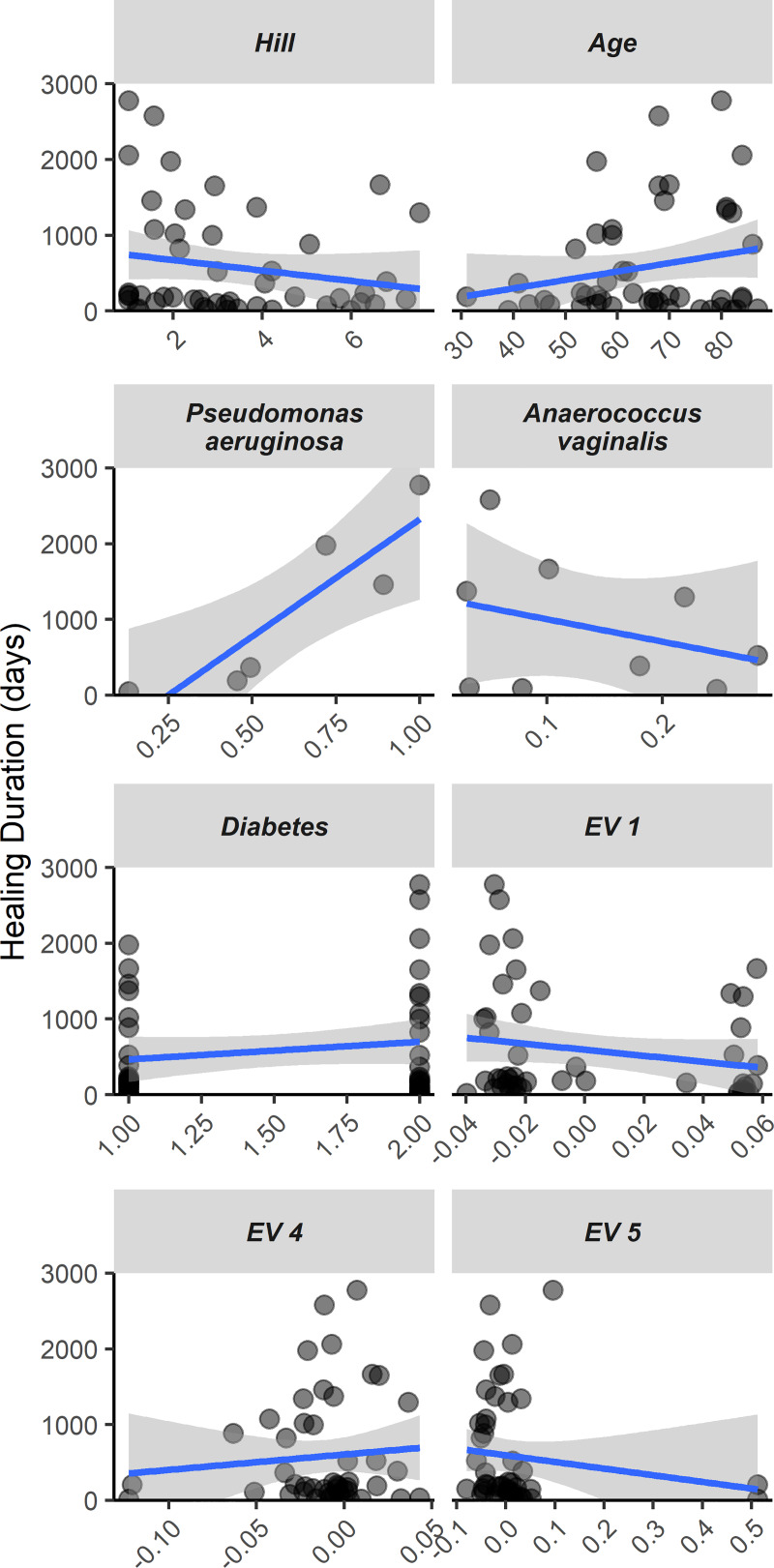
Chronic wound healing time is a function of the wound microbiome and demographics. Individual regressions of predictor variables on healing duration. Predictor variables included are those that were retained through backward selection for models of healing. For *Pseudomonas aeruginosa* and *Anaerococcus vaginalis*, only samples in which these species were observed are plotted. Variables EV1, EV4, and EV5 are population Eigen vectors encompassing genetic ancestry.

Because wound microbiomes contribute to chronicity [8–10], the relationship between wound microbiome characteristics and healing duration among individuals from both cohorts was next investigated. Wounds of 58 patients healed during the period of study, and time in days from first to last clinical visit was used to quantify wound duration (median healing time in days = 217.5, Q1-Q3 = 109–1012). Weighted least squares multiple regression with backward stepwise selection was used to determine that Hill_1_ diversity was negatively associated with wound healing (Fig 3; t = -3.21; df = 1, 54; p = 0.002; R^2^_adj_ = 0.146). Supporting that patient genetic ancestry and age additionally contribute to wound healing rate, population eigenvector (pe) 4 (t = 2.76, df = 1, 54; p = 0.008; R^2^_adj_ = 0.093) and patient age (t = 1.71; df = 1, 54; p = 0.093; R^2^_adj_ = 0.001) were the next most important variables in this model.

Because above results indicated that Hill_1_ diversity and species composition are related (Fig 1), a separate model was developed (using backward selection, as above) to investigate how relative abundances of individual species contribute to healing outcomes. This model included species with a minimum 10% prevalence (11 species) as potential predictors and the wounds in which these species occurred (n = 48). Increasing *P*. *aeruginosa* abundances were positively related with increased healing duration (t = 3.99; df = 1, 41; p < 0.001; R^2^_adj_ = 0.299), whereas *Anaerococcus vaginalis* exhibited a negative relationship (t = 2.18; df = 1, 41; p = 0.035; R^2^_adj_ ≈ 0). Patient age was more predictive in this model (t = 3.29; df = 1, 41; p = 0.002; R^2^_adj_ = 0.157), while pe 1 (t = -2.44; df = 1, 41; p = 0.019; R^2^_adj_ = 0.02), diabetes (t = 2.04; df = 1, 41; p = 0.048; R^2^_adj_ ≈ 0), and pe 5 (t = -1.89; df = 1, 41; p = 0.066; R^2^_adj_ ≈ 0) were the next most important variables. Overall, the first and second models accounted for 18.9% and 38.3% of variation in wound healing duration, respectively.

### Genes most associated with chronic wound microbiome diversity are functionally related

**Fig 4:**
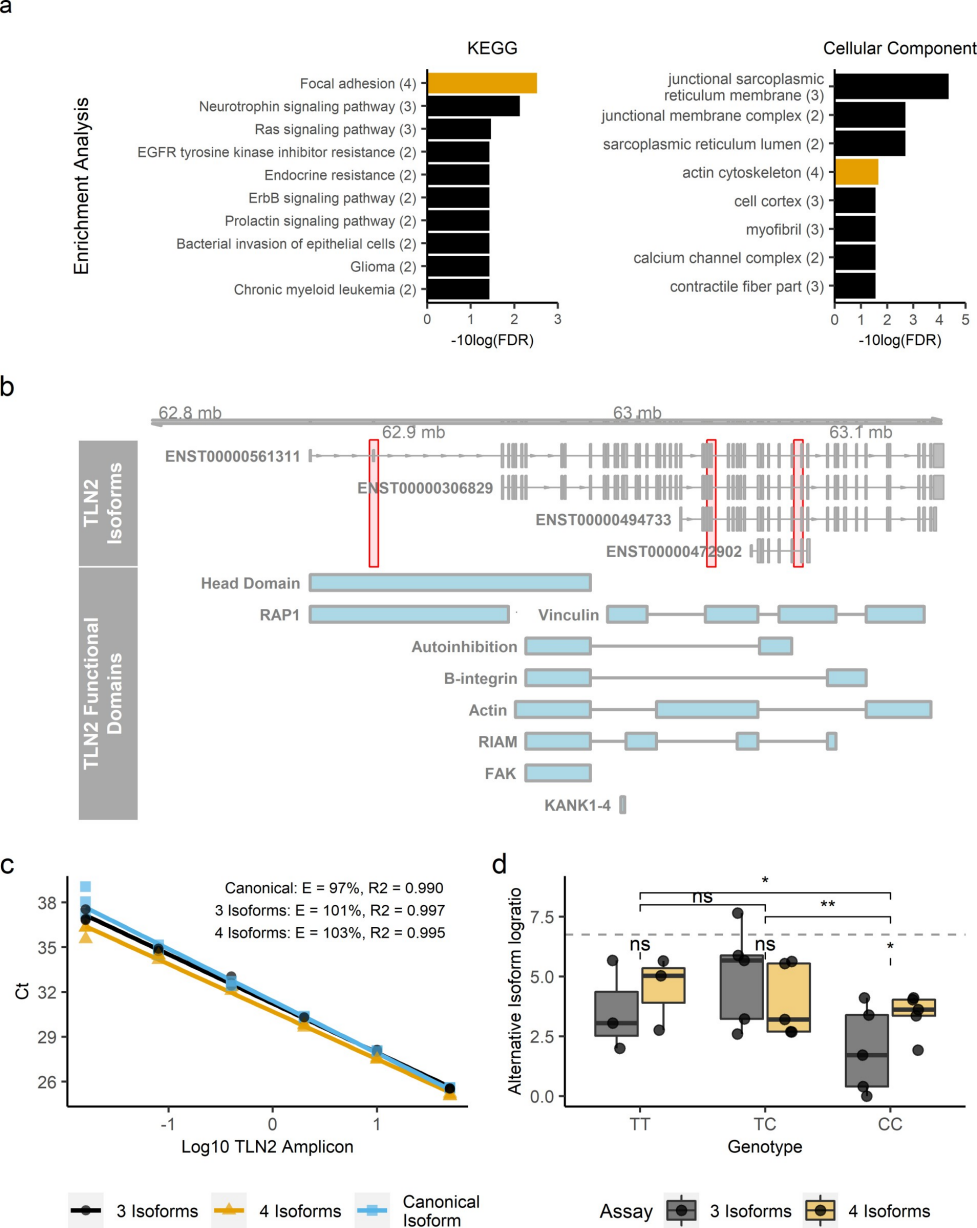
*TLN2*, functioning in cell adhesion and structure, exhibits genotype-specific alternative transcription. **a,** Select ontological terms that were enriched in this study. See S2 Table for a full list of terms. Terms involving *TLN2* are colored yellow and number of included genes (out of 15 total included in testing) per term is given in parentheses next to each term. **b,** Distribution of *TLN2* protein coding transcripts on human chromosome 15 with functional domains illustrated per Gough and Goult 2018. Red shading, from left to right denotes the transcript regions amplified from either the canonical isoform, three isoform, or four isoform assay used to compare alternative transcription by genotype (see Methods). **c,** Standard curve of RT-qPCR assays, with calculated efficiencies shown per assay. **d,** *TLN2* log isoform ratios (calculated as the log of either the three or four isoform Ct divided by the canonical Ct) were significantly associated by *rs8031916* genotype. Here, two levels of pairwise comparison are shown: above the dashed line are overall genotype comparisons, while within-genotype assay comparisons are indicated below the dashed line. Significance annotation is as follows: ns = (p>0.05), * = (p<0.05), and ** = (p<0.01).

To determine whether genes linked to suggestive/significant SNPs exhibit non-random functional relationships, a protein enrichment analysis was next performed [22]. Using genomic locations of SNPs from both cohorts a list of 15 protein coding genes was developed for analysis (*ADGRG6*, *ARHGAP24*, *FSTL4*, *KALRN*, *KANK1*, *LHX8*, *LRP1B*, *MSGN1*, *MYOZ2*, *SHC3*, *TLN2*, *TRDN*, *VASH1*, *ZNF521*, *ZNF558*). Based on a combination of co-expression data, other experimental evidence and automated text mining the list of genes was found be significantly enriched for protein-protein interactions (p < 0.001); wherein three functional linkages among the list of genes were expected by chance, 12 were identified. Moreover, multiple KEGG pathways were significantly enriched in the list of genes provided. Pathway descriptions included “focal adhesions”, “Ras signaling pathway”, “bacterial invasion of epithelial cells”, among others. UniProt, Reactome and Gene Ontology Cellular Component terms were also commonly enriched for terms/pathways describing cellular development/morphogenesis, muscle structure and function, among others (Fig 4A and see S2 Table for a full list of terms).

### Genotype at *rs8031916* explains differences in *TLN2* alternative transcription in patient wound tissue

**Fig 5:**
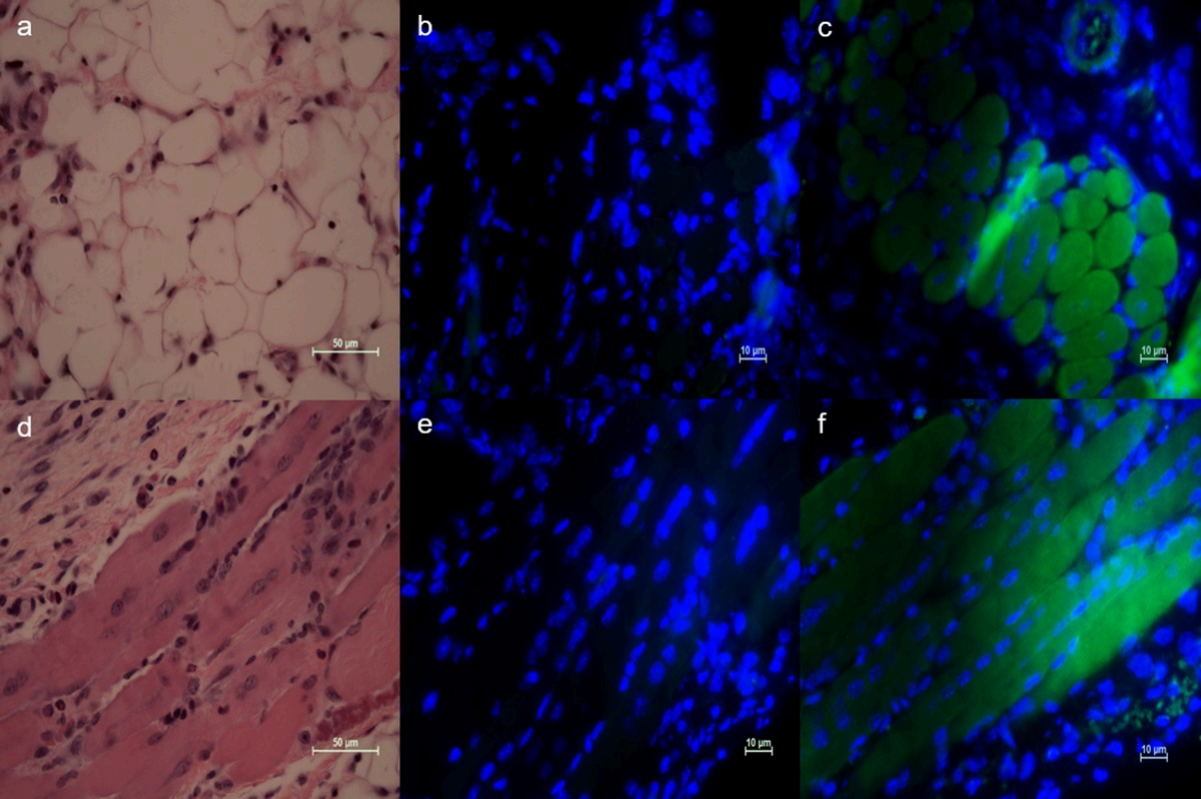
Microscopic localization of Tln2 in infected mouse wound bed. Tln2 was observed in adipocytes (a, b, c), and striated muscle (d, e, f). H&E staining (a, d), IHC negative control (b, e), IHC detection of Tln2 with Alexa Fluor 488 secondary (c, f). Nuclei were stained with DAPI. Scale bars are given for each image.

The location of *rs8031916* centrally embedded in the 454 kb human TLN2 locus and approximately 7,200 bp upstream of the shortest of four protein-coding TLN2 alternative transcripts (Fig 4B, *TLN2-202* ENST00000472902.1, GRCh38.p13) guided a prediction that the significant association of *rs8031916* with wound microbiome characteristics relates to genotype-dependent alternative transcription differences at *TLN2*. To test this prediction, *TLN2* alternative transcript expression levels in subjects’ wound bed was compared using a multiplexed reverse-transcriptase quantitative PCR assay (Fig 4C). By quantifying relative isoform expression levels, it was found that *rs8031916* genotype explained significant variation in *TLN2* isoform expression (F = 8.71; df = 2, 10; p = 0.004). More specifically, CC-genotyped individuals expressed significantly less of two intermediate length isoforms (p < 0.05, Fig 4D). However, CC-genotypes were found to express relatively more of the shortest isoform (p < 0.05, Fig 4D), and the CT and TT genotypes did not differ from each other (p > 0.05). Because wound bed biopsies were of mixed tissue it was not possible to identify the specific cell type(s) in which alternative transcription varied by genotype. A preliminary indication of *TLN2* expression in relevant cell types was provided by immunohistochemical staining of Tln2 in an infected mouse surgical excision wound model. Here, Tln2 was localized to z-lines of skeletal myocytes and was broadly expressed in adipocytes (Fig 5).

### Structural Equation Model for predicting chronic wound microbiome diversity

Drawing from both cohorts a total of 15 loci were identified to have significant/suggestive associations with chronic wound microbiome diversity (S1 Table). With the intent of developing a predictive model for chronic wound microbiome diversity, the genotypes for each associated SNP in combination with genotypes of adjacent SNPs were used to create latent variables describing each locus’ association with alpha diversity (see Methods for procedural development). The inclusion of samples from both cohorts minimized sampling variability and maximized the potential for common variance among adjacent SNPs. The resulting latent variables were used as predictors in a structural equation model (SEM), with the observation that SEM routinely outperforms observed-variable regression in model prediction as the impetus for SEM implementation. Following a backward selection procedure, six latent variables were included in the SEM that explained 52.7% of the total observed variation in alpha diversity. The six focal SNPs upon which the latent variables were based included *rs10469593*, *rs4758411*, *rs1436708*, *rs3846499*, *rs11984782* and *rs12307988* (S3 Table). Comparative Fit Index, Tucker-Lewis Index and Root Mean Square Error of Approximation for the final model was 0.971, 0.955 and 0.046, respectively. How the SEM reduced model complexity and increased explanatory power was assessed by comparison to the results of backward selection multiple regression in which all focal SNPs were initially considered as potential predictors. Whereas the SEM required six genomic loci to explain 52.7% of variation in alpha diversity, the multiple regression equation required eight SNPs and explained 10.1% less variation.

## Discussion

As predicted, chronic wound microbiome composition was significantly explained by genotype at specific loci. Although the rationale for this prediction was guided by recent findings in healthy systems, the current study provides variable loci that contribute to microbiome compositional variation in a disease state. Importantly, genotype-associated microbiome composition was also significantly related to healing, with wounds harboring lower diversity microbiomes having prolonged durations until wound closure. Genotypic effects could also help explain recently observed patient-specific immunological responses to same microbial exposure [23]. The working hypothesis that host genotype predispose individuals to infection by specific species, as well as the microbiome compositions with which species associate, invokes both environmental selection by host genotype and endogenous species interactions in deterministically shaping chronic wound microbiomes.

A notable aspect of the result that microbiome diversity was inversely related to healing time is this relationship was recovered from wound microbiomes characterized at patient’s initial clinical visit, whereas median time to wound closure was 210 days. A similar phenomenon was recently documented where the presence of anaerobic bacteria (e.g. *Peptoniphilus*) at baseline infection was associated with worse healing outcomes [24]. This relationship supports that whereas patient genetics favors colonization of certain species, priority effects [25] may influence microbiome composition, stability and wound recalcitrance. For example, *P*. *aeruginosa* was more likely to establish relative to *S*. *epidermidis* depending on genotype (Fig 1C), and wounds containing *P*. *aeruginosa* were found to have less bacterial diversity (Fig 1D) and longer healing times (Fig 3). Complementary to these findings is the observation from recent work examining wound microbiome trends receiving topical multi-antibiotic therapy in which *Pseudomonas* dominated wound communities exhibited increased temporal stability in comparison to Staphylococcal infections [11].

Comparison of models for healing duration help shed light on the relative importance of individual species effects versus emergent community properties on patient outcomes (Fig 3). The observation that variance uniquely accounted to species relative abundances (~29%, primarily due to *P*. *aeruginosa*) was almost double that accounted for by microbiome diversity alone (~15%) suggests that the presence of consequential species, and not community composition, is the primary determinant of healing outcome. While this comparison is informative, the relationship between healing and diversity was observed despite only 12.5% of wounds containing *P*. *aeruginosa*, potentially indicating wound healing differences also due to emergent community properties. Previous work demonstrates how individual species and communities can both be consequential to pathogenicity. For example, the retention of type 3 secretion system functionality in *P*. *aeruginosa*, which is used for host substrate interaction, was found to be a more critical requisite for infection than pathways relevant to microbial interaction [26]. Conversely, synergy between pathogens has been demonstrated to increase biofilm pathogenicity [10, 27]. The synthesis of existing information indicates that patient genetics, intrinsic microbial properties which promote growth and infection [26], and interspecific interactions [8] all shape wound microbiomes, host responses and healing outcomes.

Patient genotype at *TLN2* intronic variant *rs8031916* explained significant variation in both alpha and beta diversity (Fig 1), as well as the relative abundances of the two species most observed in mono-infections in this particular patient population, *P*. *aeruginosa* and *S*. *epidermidis* [3]. Talin is a fundamental component of focal adhesion (FA) formation where it is involved in f-actin polymerization [28], integrin activation [29], FA stability [30], and is also required for the assembly of cortical microtubule stabilizing complexes [31]. Talin regulation is achieved through direct interactions with F-actin, β-integrins, VCL, KANK1, among others [31, 32]. FA are essential for cell contact, movement and signaling, and aberration of FA by loss or altered talin production results in reduced cell mobility [30], attachment [33], and healing [34]. In addition, FA are exploited by microorganisms during cellular attachment and invasion [35, 36], and talin has been directly implicated in this process [37]. Here, comparison of relative expression levels of *TLN2* alternative transcripts in wound bed biopsies showed that subject genotype at *rs8031916* also explained significant differences in *TLN2* alternative transcript expression, and these transcripts differ in their inclusion of β-integrin, VCL, F-actin and KANK1 binding motifs ([31, 32], S1 File, Fig 4B). The emergent working hypothesis is that *TLN2* alternative transcript production influences focal adhesion dynamics, such as size and stability, which may shape bacterial exploitation of FA, may alter cell mobility and healing, or some combination of the two. Quantifying the influence of these processes should be investigated in the future using techniques including fine mapping, FA imaging in different genetic backgrounds, absolute isoform quantitation, and bacterial attachment and cell migration assays. However, alternative transcription by tissue type is currently ambiguous; wound bed biopsies were of mixed tissue type and the limited survey of infected mouse wound bed highlighted *TLN2* expression in both adipocytes and myocytes, and other yet-to-be surveyed cell types may also be relevant. Tissue-specific alternative transcription could be characterized with a follow-up transcriptome wide association study [38] to identify candidate tissues, proceeded by isoform expression measurements in pure culture in combination with crispr-mediated mutagenesis of candidate SNPs.

The idea that genetically determined FA phenotypic variation selects for microbial colonization is supported by work in which FA-relevant loci were associated with the persistence of *S*. *aureus* in healthy sinuses [18]. Moreover, other recent work in wound infiltrated neutrophils showed that perturbation of *MiR-142*, which influences FA via regulating *Rac1* and *RhoA* protein synthesis, led to increased bacterial burden and delayed healing [39]. Here, the importance of FA phenotypic variation in wounds is further supported by broader consideration of SNPs that were significantly or suggestively associated. The statistical enrichment of protein interactions among candidate loci was influenced from inclusion of genes known to function in FA or otherwise in cytoskeletal and membrane interactions (Fig 4A, S2 Table). For example, KANK1 was among candidate loci, and is known to interact directly and essentially with TLN2 to link FA [40] and cortical microtubule complexes [31]. Notably, the TLN2 KANK-binding domain is missing from the shortest TLN2 alternative transcript which differed in expression depending on *rs8031916* genotype. Whereas FA relationships stand out among candidate loci, further survey indicates additional cellular processes also warranting additional investigation. For example, while genetic variation at *ZNF521* may be consequential to BMP-signaling [41, 42] which influences cell migration and healing [43–45], *ZNF521* regulation of B cell maturation is also an important component of the immune response [42, 46].

Lastly, a structural equation model using patient genotype as indicators was developed that accounted for more than half of the observed variance in microbial diversity, a metric associated here with healing and recently with the continuum between wounds and healthy skin [6, 12]. The prospect that patient genetics shape chronic wound microbiome composition highlights a new opportunity to identify biomarkers for clinically relevant predictive models. For example, such models could inform risk for development and persistence of specific infections. Given that wound persistence is associated with development of multiple drug resistant pathogens [9], such models could identify cases where aggressive targeted therapy is warranted at onset. In general, expanded mbGWAS and experimental validation should reveal additional genetic variants and corresponding phenotypes determining chronic infection.

## Methods

### Ethics statement

Human study protocol was executed after obtaining informed written consent from adult patients for study as approved under Western Institutional Review Board protocol (Protocol number: 20171819). This study was carried out in strict accordance with the recommendations in the Guide for the Care and Use of Laboratory Animals of the National Institutes of Health. The protocol was approved by the Institutional Animal Care and Use Committee of Texas Tech University Health Sciences Center (protocol number 07044). Mice were anesthetized using 0.02 ml per gram weight of Nembutal (pentobarbital sodium, 5 mg/ml) and euthanized with 150 μl of Fatal-Plus solution (pentobarbital sodium, 390 mg/ml).

### Sample collection

Subjects recruited into this study were drawn from patients visiting the Southwest Regional Wound Care Clinic (Lubbock, TX, USA) for treatment of chronic wound infections. Patients were candidates for inclusion on the basis that they presented with a lower extremity wound, physician-determined wound infection which had been characterized at initial visit, and were adults who consented to study conditions. Infection was determined by the accumulation of excess necrotic material and persistent exudate on the surface of the wound, in accordance with established guidelines [47]. Subjects were not selected based on wound type (i.e. diabetic ulcer, venous ulcer, decubitus ulcer) because previous work has shown microbiome composition is not significantly influenced by wound type [3]. Following written consent, a single buccal swab was collected from each subject. Manually homogenized wound microbiome debridement from each patient’s wound was also collected as previously described [3]. Wound debridement samples were sent to MicroGenDX (Lubbock, TX, USA) for bacterial community profiling (see below). Buccal swabs were immediately placed in liquid nitrogen and transferred for cryogenic archival in the Wolcott Wound Care Research Collection at the Natural Science Research Laboratory, Museum of Texas Tech University. Ninety-four buccal samples were initially retrieved from this archive and defined as the exploratory cohort. Following mbGWAS (see below), an additional 96 buccal swabs were selected and defined as the experimental cohort. Buccal samples were selected for both cohorts on the basis that they were collected from subjects who had received wound microbiome community profiling on their initial clinical visit. Healing duration was defined from day of first visit to the wound clinic until day that physician determined complete closure of wound. Ethnic breakdown for those included in final analysis for the exploratory and experimental cohorts was 6% and 11% Black, 33% and 25% Hispanic, and 61% and 64% White, respectively.

### Wound microbiome profiling

Only wound samples from the first clinical visit (i.e., before clinic treatment effects) were included for analysis. Depending on the date of initial clinic visit, the relative abundance bacterial composition of wound samples was characterized using either a 454 Titanium Instrument (454 Life Sciences, Roche, Brandord, CT, USA) as previously described [3, 11] or using an Ion Torrent PGM (Thermo Fisher Scientific, Waltham, MA, USA) at MicroGenDX (Lubbock, TX, USA). Clinical repeatability and congruence in community profile reporting between instruments was previously validated during laboratory certified authorization professional (CAP) certification of this facility. The protocol for PGM 16S sequencing has not been previously reported and is described here in brief. Total DNA was extracted as previously reported [3, 11]. PCR reactions used primers 28F (GAGTTTGATCNTGGCTCAG) and 388R (GCTGCCTCCCGTAGGAGT) with Quanta AccuStart II Tough Mix (Quanta bio, Beverly, MA, USA). PCR reactions were conducted on ABI Veriti thermocyclers (Applied Biosystems, Carlsbad, CA, USA) with a thermal profile consisting of 5-minute denaturation step at 95°C, 35 cycles of 94°C for 30 seconds, 52°C for 40 seconds, and 72°C for 60 seconds, and a final extension step of 72°C for 10 minutes. PCR products were grouped equal molar and selected by size in two rounds using Agentcourt AMPure XP (Beckman Coulter, Indianapolis, Indiana, USA) in a seven-tenths ratio of AMPure to product. Quantification of each group was carried out using a Qubit 2.0 fluorometer (Thermo Fisher Scientific, Waltham, MA, USA). Downstream emulsion PCR, recovery, enrichment and sequencing followed PGM manufacturer protocols. Bioinformatic processing of sequences was the same as that previously described and employed a 1% within-wound minimum relative abundance threshold [3].

### Subject genomic DNA extraction

Buccal swabs were transferred to the laboratory in liquid nitrogen, briefly thawed and 500 μL of Sterile Longmire’s Solution (100 mM Tris, 100 mM EDTA, 10 mM NaCl, 0.5% SDS) was added to each sample vial. Extraction of genomic DNA was accomplished using Gentra PureGene Buccal Cell Kits (Qiagen, Gaithersburg, MD, USA) incorporating 65°C lysis for 1 hour, RNase A treatment at 37°C for 30 minutes, and a final elution in HyClone HyPure Molecular Biology Grade Water (GE Healthcare Life Sciences MA, USA).

### Subject genome fingerprinting

All DNA samples were concentrated, if necessary, and normalized to the range of 10–50 ng/μL. Samples were genotyped according to the manufacturer’s protocol using the Infinium Global Screening Array -24 v2.0 microarray (Illumina, San Diego, CA, USA), which includes 665,608 markers focused on genome-wide tag-SNP variant coverage and clinically relevant research loci. Data were collected using the iScan System (Illumina), and preliminary processing/analysis (i.e., normalization, SNP clustering, genotype calling, call rate calculations, array-based QC) were conducted using GenomeStudio v.2.0 (Illumina). Plink file sets were exported using the PLINK Input Report Plug-in v.2.1.4 within GenomeStudio. Quality control (QC) filtering was performed in accordance with Anderson et al., 2010. Briefly, individual level QC was performed to remove individuals (1) based on missingness (>0.09) and observed heterozygosity deviations (±3 SD from mean); (2) who exhibited a high degree of allele sharing/relatedness using an Identity-by-Descent threshold of 0.1875; and (3) with a high degree of ancestral divergence assessed via principal component analysis using SNPRelate [48]. Marker-level filtering was performed to remove markers with low genotyping rates <0.05; and minor allele frequency <0.05. Principal component analysis via SNPRelate was performed again on the post-quality control dataset to generate correct eigenvectors for population structure correction in subsequent analyses.

### GWAS-microbiome comparison

For genomic associations microbiomes were summarized as alpha diversity represented as effective number of species (i.e. Hill_1_ Numbers [49] expressed as the exponential function of Shannon Diversity, which are more appropriate for linear models [50]). PLINK v1.9 [21] was used to merge phenotype and covariate data with genotypes. Associations between SNPs and alpha diversity were assessed using PLINK linear models. Sex, diabetic status, age, and the first five eigenvectors from a PCA of the SNP data (continuous variables primarily describing genetic ancestry) were included based on distribution of p-values [20], visualized using the R package qqman [51]. The threshold for statistical significance was defined as p < 1.57 x 10^−7^ (0.05/317,553 SNPs considered), and the threshold for suggestive loci was defined as two orders of magnitude greater (p < 1.57 x 10^−5^). For the exploratory cohort p-values for eight SNPs fell within the suggestive region (S1 Table) and were subsequently considered candidate loci. Subsequent association testing of SNPs in the experimental cohort was initially confined to these candidate loci. Following statistical testing of candidate loci, a full list of suggestive/significant SNPs from the experimental cohort was developed the same as for the exploratory cohort and were used for specific downstream statistical tests (i.e. protein-protein interactions, functional enrichment, and predictive modeling (see below)).

### Patient genotype-species relative abundance relationships

Following confirmation from analysis of the experimental cohort that *TLN2* and *ZNF521* were associated with wound microbiome alpha diversity, data from both cohorts were combined to assess the relationship between an individual’s genotype at these loci and the relative abundance of bacterial species. To limit the number of statistical comparisons being made, tests were only made for bacterial species that were present in at least 10% of wound microbiomes (11 bacterial species). The covariates described above were included as explanatory variables in the MANOVA to assess the relationship between bacterial species abundance and subject genotype.

### Patient genotype-chronic wound microbiome beta diversity relationships

How patient genotype at *TLN2* or *ZNF521* explained variance in patient wound microbiome composition was next quantified. This analysis employed a permutational analysis of variance [52] that incorporated samples from both cohorts, covariates and beta diversity summarized as Bray-Curtis dissimilarities. Distance-based redundancy analysis was used to visualize sample relationships and correlations of bacterial species relative abundances with the first two components of variation were used to define biplot vectors.

### Correlation network among bacterial species

To limit the sparsity of the community matrix, bacterial species present in at least 5% of patient wounds were included for canonical Pearson correlation. R package geomnet was used to construct an interaction network using associations greater that an absolute value of 0.1.

### Microbiome-wound duration relationship

From both cohorts, a total of 58 patient wounds had healed by the time of statistical analysis for this study. For these samples multiple Weighted Least Square (WLS) regression was used to assess the relationship between alpha diversity at initial sampling and healing duration; WLS regression was used due to the unequal variance structure between Hill_1_ and healing duration (i.e., variance in healing rate decreased as Hill_1_ increased; Breusch-Pagan test for heteroscedasticity, BP = 15.4, df = 3, p = 0.001). Relationships between healing and species relative abundances were also assessed but in a separate model due to the significant relationship between alpha diversity and individual species presence. For this model, ordinary least squares regression was used and candidate predictive taxa were defined as those exhibiting a minimum 10% prevalence across the 58 wounds. Patients with wounds containing species above this threshold were included. For both models, backward selection was used to determine explanatory variable inclusion starting from all potential explanatory variables, which included covariates as in the linear mbGWAS.

### Immunohistochemistry of *TLN2* in Mouse Wound Model

Given the consistently recovered relationship of *TLN2* with different aspects of chronic wound microbiome composition, a mouse wound model was used to evaluate if Tln2 protein expression could be detected in an infected wound bed. The mouse chronic wound infection model used here has been previously described [53, 54]. Briefly, 8-week old female Swiss-Webster mice (Charles River Laboratories, Wilmington, MA) were anesthetized by intraperitoneal injection of sodium pentobarbital and administered full-thickness, dorsal excisional skin wounds to the level of the panniculus muscle. The wounds were then covered with a semipermeable polyurethane dressing (Opsite dressing; Smith & Nephew), under which 10^4^ *Pseudomonas aeruginosa* (PA01) bacterial cells were injected into the wound-bed. Bacterial biofilm formation was allowed to proceed for 7 days, after which the wound beds were excised, fixed in 10% buffered formalin, and embedded in paraffin.

Immunohistochemical staining of Tln2 took place after deparaffinizing the tissue in three 5-minute washes in xylene, two 5-minute washes in absolute ethanol, one 3-minute wash with 95% ethanol, and one 3-minute wash with 70% ethanol. Antigen retrieval was performed by flooding with 20 ug/ml proteinase K for 15 minutes, and near-boiling (95°) in 10 mM sodium citrate buffer for 12 minutes. Tln2 was localized with a combination of 2 μl/ml Rabbit anti-TLN2 antibody (Cat: R32413, NSJ Bioreagents, San Diego, CA) incubated overnight at 4°C, followed by 2 μl/ml Donkey anti-rabbit Alexa Fluor 488 (Cat: A21206, Life Technologies, Eugene, OR) antibody incubated for 1 hour at room temperature. Controls were performed by staining adjacent tissue slices with the secondary antibody alone. All sections were imaged with a Nikon Eclipse 80i fluorescence microscope, under a 100x oil immersion objective.

### Protein-protein interaction and functional enrichment

A list of genes corresponding to all gene-associated SNPs from both cohorts with p-values smaller than the suggestive cut-off were included in a search against string-db ([22], accessed February 05, 2019) invoking a minimum required interaction score of 0.15 to indicate enrichment for interacting proteins in the provided list. Pathway-term enrichment among input proteins was evaluated using a false-discovery rate p-value correction. The human genome was used as the statistical background for enrichment tests.

### *TLN2* alternative transcript expression assay

To investigate the effect of genotype at *rs8031916* on *TLN2* alternative transcription, a multiplexed reverse-transcriptase quantitative PCR (RT-qPCR) assay was developed. The assay was designed such that multiplexed amplification of different regions of the *TLN2* open-reading frame expressed in different transcripts allowed comparison of relative expression levels. To this end, cDNA sequences were downloaded from ensembl.org representing the four known *TLN2* alternative transcripts and aligned using MUSCLE [55]. Three regions of aligned cDNA were selected as targets for assay development including 1) a region only expressed in the canonical (it is also the longest) *TLN2* isoform (ENST00000561311.5), 2) a region expressed in the three longest isoforms (ENST00000561311.5, ENST00000636159.1, ENST00000494733.5), and 3) a region expressed in all four isoforms (those mentioned above as well as ENST00000472902.1). A primer combination targeting a region expressed only in ENST00000561311.5 and ENST00000561311.5 was not created due to lack of acceptable primer binding sites. Fig 4B provides a graphical representation of the *TLN2* locus and the approximate locations targeted by assays.

A *TLN2* standard, which was used to validate the RT-qPCR assays, was created by amplifying a 4.9 kb region of *TLN2* from cDNAs created from total human muscle RNA (Takara Bio, Mountain View, CA). Following manufacturer recommendations, SuperScript IV VILO MasterMix (ThermoFisher, Waltham, MA) was used to create cDNAs, amplification was accomplished using Phire Hot Start II (ThermoFisher, Waltham, MA; 98°C for 2 min; 30 cycles of 98°C 20 s, 56°C 20 s, and 72°C 1 min 45 s), and the PCR product was purified by elution through QIAquick columns (30 uL Buffer EB; Qiagen, Valencia, CA). A serial dilution (1:5) of the final product was used as template to create the multiplexed standard curve (Fig 4C). Standard curve RT-qPCR reactions were carried out following IQ multiplex powermix protocol (Bio-Rad Laboratories, Hercules, CA) with cycling conditions as follows: 95°C for 2 min; 40 cycles of 95°C 10 s and 56°C 45 s. All primer sequences (S4 Table) were selected using the Primer3 [56] tool and manufactured by IDT (Integrated DNA Technologies, Coralville, IA)

RNA was extracted from 50(±5) mg wound biopsies representing 13 individuals using the Monarch Total RNA Miniprep kit (New England BioLabs, Ipswich, MA) and normalized to 100 ng total RNA going into VILO cDNA synthesis reactions. Wound biopsies were often highly heterogeneous in their composition of apparent muscle, skin, and adipose tissue. cDNA products were immediately used for RT-qPCR reactions with the conditions described above for standard curves. Inter-sample expression comparisons were enabled by calculating the isoform ratios as the Ct values of either the three or four isoform assays to the Ct value of the assay only amplifying the canonical isoform. Log-transformed ratios were analyzed by multi-factor ANOVA, accounting for variation by subject genotype, assay, sample, as well as genotype-assay interaction. Genotype pairwise comparisons were conducted by Tukey’s HSD test, and within genotype and assay pairwise comparisons using paired T-tests.

### Development of a Structural Equation Model to predict chronic wound microbiome diversity

Structural Equation Modeling (SEM) is a general method for estimating and modeling latent variables. Latent variables are typically unobserved or unmeasurable constructs that cannot be directly evaluated, which are instead inferred indirectly through observed indicators. SEM has found recent application in genomics research [57]. Here, a novel application of SEM is employed in which latent variables are used to summarize the common variance of multiple SNPs within a genomic region. The latent variable for each genomic region is estimated from the correlations of a focal SNP (i.e., each of 15 suggestive/significant SNPs identified in mbGWAS from both cohorts) with genomically-proximal SNPs that also segregated with the outcome (i.e. alpha diversity), but to a lesser extent due to genomic recombination or reduced effects on the outcome. The predictive capability on wound microbiome diversity of resulting latent variables were then assessed through a model building routine. In detail, genomically-proximal SNPs to include as candidate indicators were selected by identifying the elbow in the distribution of p-values (from mbGWAS). For this, SNPs located either side of each focal SNP were sorted by increasing p-value. The elbow in the distribution of p-values among 20 proximal SNPs was identified as the absolute value of the difference in p-values of sorted adjacent SNPs. Manual adjustment of elbow locations, which had the effect of reducing the number of retained loci, was based on visual inspection of ranked p-values.

Remaining SNPs were then integer coded based on correlation with the outcome. Alternative integer coding at the biallelic SNPs (e.g. AA, AG, GG) implied either first allele dominance (0 0 1), heterozygote dominance (0 1 0), second allele dominance (1 1 0) or additive effects in either direction (0 1 2 or 2 1 0). In the case of loci for which only two genotypes were observed, directionality was coded in both directions (i.e. 0 1 or 1 0). For each SNP, Pearson correlations with alpha diversity were calculated for each alternative integer coding, and the coding with the strongest correlation was retained. Following integer coding, correlations amongst integer-coded SNPs within each genomic region were calculated. From these correlation matrices, the number of candidate-indicator SNPs was refined by retaining SNPs that typically exhibited correlations with the focal SNP greater than 0.40 and had a correlation greater than 0.20 with another SNP passing this criterion [58, 59]. When candidate-indicator SNPs had correlations greater than 0.60, a new candidate-indicator was created by averaging their values. The final list of indicators per locus was defined by up to three SNPs based on strength of correlation with the outcome.

The relationship of each latent variable (representing each locus) with alpha diversity was assessed using Confirmatory Factor Analysis (CFA) implemented in the R package lavaan [60]. The R^2^ value of each CFA was compared to that obtained from a general linear model (GLM). In instances where CFA outperformed GLM the latent construct was retained, otherwise the genomic region was represented simply by the original integer-coded genotype call from the focal SNP.

The final set of variables were specified as predictors of alpha diversity in an SEM that was iteratively reduced by removing predictors that did not explain a significant unique component of variation through backward stepwise selection. Performance of the SEM approach was benchmarked by comparison to a multiple regression equation also developed through backward selection with focal SNPs serving as predictors. SEM model fit was assessed with the Comparative Fit Index (CFI), Tucker-Lewis Index (TLI) and Root Mean Square Error of Approximation (RMSEA). The scripts used for all statistical analysis are available at https://github.com/genotyper/mbGWAS_wounds.

## Supporting information

S1 Fig Q-Q plot visualizing distribution of p-values following exploratory cohort mbGWAS. (TIFF)

S2 Fig Manhattan plot depicting results of exploratory mbGWAS on Hill_1_ Diversity. (TIFF)

S3 Fig LocusZoom plot for *rs8031916*. (PDF)

S4 Fig LocusZoom plot for *rs7236481*. (PDF)

S1 Table Results of mbGWAS on Hill_1_ Diversity. (CSV)

S2 Table Protein enrichment summary. (CSV)

S3 Table Summary of SNPs used in SEM. (DOCX)

S4 Table Primers used during RT-qPCR validation and experiment. (DOCX)

S1 File Talin transcript summary of domains. (TXT)
