# Supplementary figures and images for: Patient genetics is linked to chronic wound microbiome composition and healing
Source: PLoS Pathog. 2020 Jun 18;16(6):e1008511. doi: 10.1371/journal.ppat.1008511 (PMC7302439; doi:10.1371/journal.ppat.1008511)

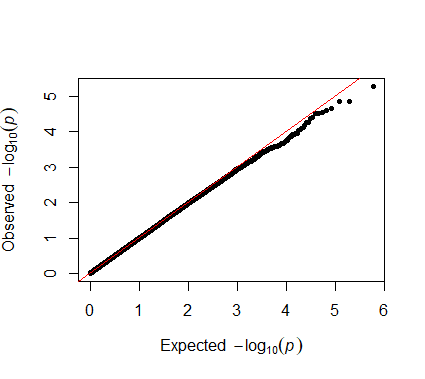

Supplement: S1 Fig — (TIFF) [file ppat.1008511.s001.tiff]

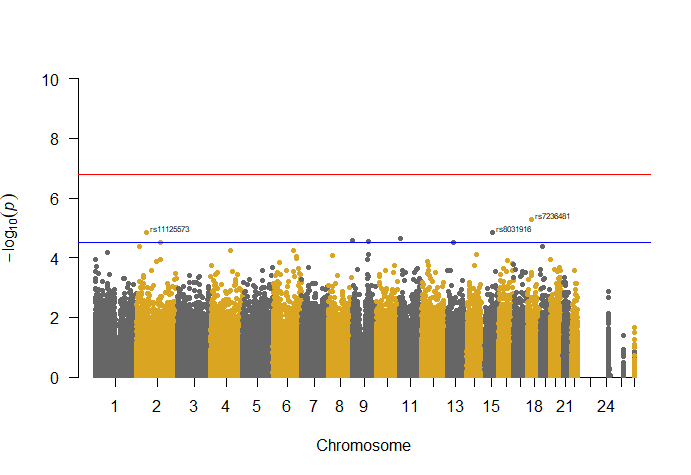

Supplement: S2 Fig — (TIFF) [file ppat.1008511.s002.tiff]
