# Supplementary material for: Patient genetics is linked to chronic wound microbiome composition and healing
Source: PLoS Pathog. 2020 Jun 18;16(6):e1008511. doi: 10.1371/journal.ppat.1008511 (PMC7302439; doi:10.1371/journal.ppat.1008511)
Supplement: S3 Fig — (PDF) [file ppat.1008511.s003.pdf]

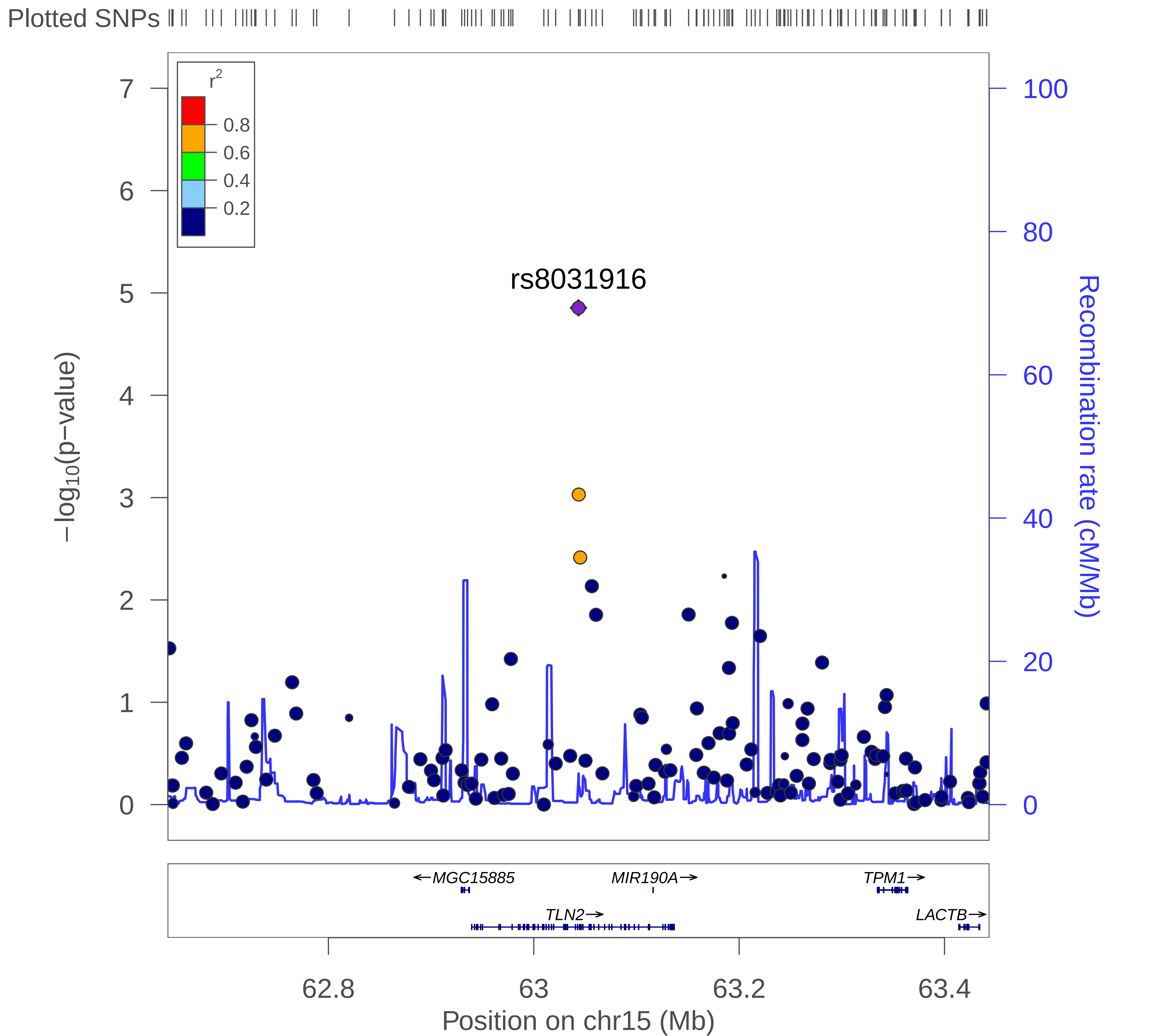

Plotted SNPs
r2
0.8
0.6
0.4
0.2
rs8031916
−log10(p−value)
Recombination rate (cM/Mb)
0
1
2
3
4
5
6
7
0
20
40
60
80
100
MGC15885
MIR190A
TPM1
TLN2
LACTB
62.8
63
63.2
63.4
Position on chr15 (Mb)

date:	Thu Feb 14 05:35:14 2019
build:	hg19
display range:	chr15:62643547−63443547 [62643547−63443547]
hilite range:	0 − 0 [0 − 0]
reference SNP:	chr15:63043547
number of SNPs plotted:	133
min P:	1.4E−5 [chr15:63043547]
max P:	9.98E−1 [chr15:63009804]

Make more plots at http://csg.sph.umich.edu/locuszoom/
