# Supplementary material for: Patient genetics is linked to chronic wound microbiome composition and healing
Source: PLoS Pathog. 2020 Jun 18;16(6):e1008511. doi: 10.1371/journal.ppat.1008511 (PMC7302439; doi:10.1371/journal.ppat.1008511)
Supplement: S4 Fig — (PDF) [file ppat.1008511.s004.pdf]

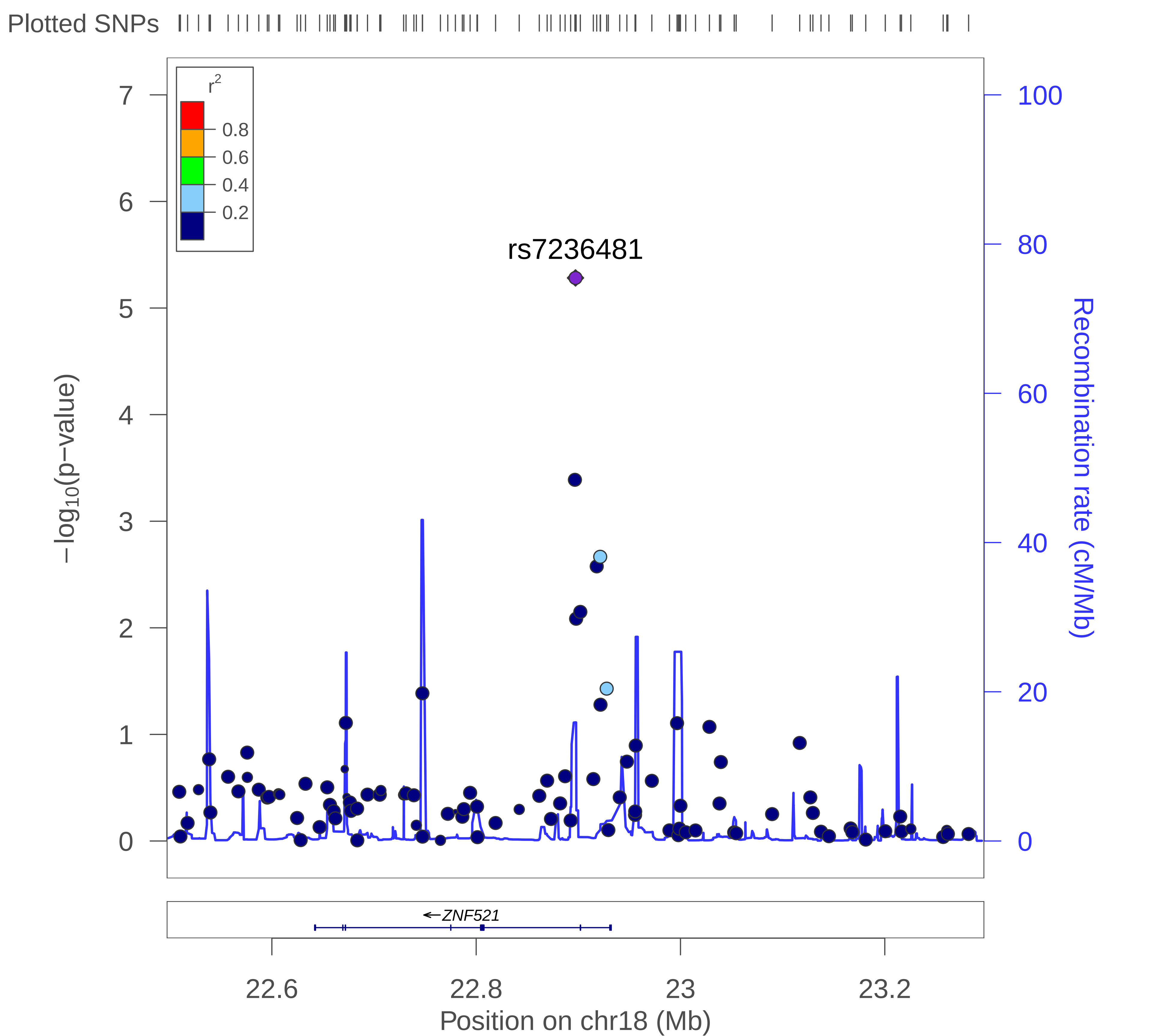

Plotted SNPs
r2
0.8
0.6
0.4
0.2
rs7236481
−log10(p−value)
Recombination rate (cM/Mb)
ZNF521
22.6
22.8
23
23.2
Position on chr18 (Mb)

date:	Thu Feb 14 05:48:01 2019
build:	hg19
display range:	chr18:22497237−23297237 [22497237−23297237]
hilite range:	0 − 0 [0 − 0]
reference SNP:	chr18:22897237
number of SNPs plotted:	102
min P:	5.22E−6 [chr18:22897237]
max P:	9.85E−1 [chr18:22683632]

Make more plots at http://csg.sph.umich.edu/locuszoom/
