# Supplementary material for: Patient genetics is linked to chronic wound microbiome composition and healing
Source: PLoS Pathog. 2020 Jun 18;16(6):e1008511. doi: 10.1371/journal.ppat.1008511 (PMC7302439; doi:10.1371/journal.ppat.1008511)
Supplement: S3 Table — (DOCX) [file ppat.1008511.s007.docx]

S4 Table. Summary of SNPs used for the construction of latent variables included in the final SEM predicting chronic wound microbiome alpha diversity. Focal SNP = SNPs that were initially identified through mbGWAS, Additional indicator SNPs = SNPs adjacent to the respective focal SNPs, the genotypes of which were used to help formulate the corresponding latent variable (NA if the focal SNP was used alone; an underscore between two SNPs indicates that the average of the integer-coded genotypes of the respective loci served as the indicator), Known gene association = corresponding focal SNP’s physical gene association followed by its genomic location in the format chromosome:base position (based on [insert genome build version]) , Individual *R^2^* = explanatory power of each latent variable based on preliminary models with only one predictor included per model.

| Focal SNP | Additional indicator SNPs | Known gene association | Individual *R^2^* |
| --- | --- | --- | --- |
| rs201276730 | rs10469593 + rs10496839 + rs11894060_rs201276730 | LRP1B (2:141041879) | 0.042 |
| rs4758411 | rs2682111 | LOC101927825 (11:6365704) | 0.095 |
| rs1436708 | NA | None (13:55818627) | 0.041 |
| rs3846499 | rs32483 + rs6881771 | none (5:50254200) | 0.093 |
| rs11984782 | rs11989865 | none (8:78683460) | 0.068 |
| rs12307988 | rs7137605_exm2267480 | LOC101929974 (12:131974235) | 0.202 |
