# Supplementary material for: Patient genetics is linked to chronic wound microbiome composition and healing
Source: PLoS Pathog. 2020 Jun 18;16(6):e1008511. doi: 10.1371/journal.ppat.1008511 (PMC7302439; doi:10.1371/journal.ppat.1008511)
Supplement: S4 Table — (DOCX) [file ppat.1008511.s008.docx]

**S4 Table. Primers used during RT-qPCR validation and experiment.**

| **Assay** | **Forward Primer** | **Reverse Primer** | **Probe** |
| --- | --- | --- | --- |
| TLN2_validation | GGA TGA GAA GAC CAA GGA AGT G | CCG AGA GCA CCA AGG AGA |  |
| TLN2_209 | CAC ACT GGA TTT TGG GGA GT | CAA TGT AGC CTG CAA TCA GC | /5Cy5/AAC CAC CGA /TAO/ GGG AGA GCA GA/ 3IAbRQSp/ |
| TLN2_3iso | CGA GAC TGT GAA GGG GAT GT | CAT CGA TTC ACC CAG AAC CT | /5HEX/CTG CAT TGA /ZEN/ GAG TGT GAT GGA/ 3IABkFQ/ |
| TLN2_4iso | AAC GAA GCT GCC AGT GAA GT | TTC TGG AGG AGT GCC TTC AT | /56-FAM/ TGC AGA AGC /ZEN/ CAT GAG CAA GC/ 3IABkFQ/ |
